# Genetic structure of the European hedgehog *(Erinaceus europaeus)* in Denmark

**DOI:** 10.1371/journal.pone.0227205

**Published:** 2020-01-17

**Authors:** Sophie Lund Rasmussen, Jeppe Lund Nielsen, Owen R. Jones, Thomas B. Berg, Cino Pertoldi

**Affiliations:** 1 Department of Biology, University of Southern Denmark, Odense, Denmark; 2 Department of Chemistry and Bioscience, Section of Biotechnology, Aalborg University, Aalborg, Denmark; 3 Interdisciplinary Centre on Population Dynamics (CPop), Department of Biology, University of Southern Denmark, Odense, Denmark; 4 Naturama, Svendborg, Denmark; 5 Aalborg Zoo, Aalborg, Denmark; Universitat Pompeu Fabra, SPAIN

## Abstract

**Objectives:**

Low genetic diversity can lead to reduced average fitness in a population or even extinction. Preserving genetic connectivity across fragmented landscapes is therefore vital to counteract the negative consequences of genetic drift and inbreeding. This study aimed to assess the genetic composition and consequently the conservation status of a nationwide sample of European hedgehogs (*Erinaceus europaeus*) in Denmark.

**Methods:**

We applied an adaptation of the genotyping by sequencing (GBS) technique to 178 individuals from six geographically distinct populations. We used a Bayesian clustering method to subdivide individuals into genetically distinct populations. We estimated individual observed (iH_O_), observed (H_O_), and unbiased expected (uH_E_) heterozygosity, inbreeding coefficient (F_IS_), percentage of polymorphic loci (P%) and tested for deviations from Hardy-Weinberg equilibrium (HWE). We used linear models to test for potential anthropogenic effects on the genetic variability of hedgehogs with iH_O_, uH_E,_ P% and F_IS_ as response variables, and assessed the demographic history of the population.

**Results:**

The Danish hedgehog population is composed of three genetic clusters. We found a mean P% of 54.44–94.71, a mean uH_E_ of 0.126–0.318 and a mean H_O_ of 0.124–0.293 in the six populations. The F_IS_ was found to be significantly positive for three of the six populations. We detected a large heterogeneity of iH_O_ values within populations, which can be due to inbreeding and/or fragmentation. F_IS_ values decreased with increasing farmland density, but there was no significant association with human population or road density.

**Conclusions:**

We found a low level of genetic variability and evidence for genetic substructure and low effective population size, which are all consequences of habitat fragmentation. We failed to detect signs of a recent population bottleneck or population increase or decline. However, because the test only identifies recent changes in population size, we cannot reject the possibility of a longer-term decline in the Danish hedgehog population.

## Introduction

The western European hedgehog (*Erinaceus europaeus*, hereafter referred to as “hedgehog”), is a widely-distributed nocturnal generalist predator species found in a range of habitats in the British Isles, New Zealand and continental Europe, from Iberia and Italy northwards into Scandinavia [1, 2]. Despite their wide geographic distribution, the species is feared to be in decline, based on research conducted at collected at national and local scale from several western European countries including the UK, Belgium, the Netherlands, Sweden and Germany [3–12]. It is suggested that the decline is more severe in the rural than urban areas [5, 7]. Although we lack population trend data for Denmark, it is likely that the situation is similar because of comparable habitat fragmentation, landscape structure, farm management practices and climate across north-western European countries.

Potential drivers of this decline include habitat loss and fragmentation, which are a great concern in terrestrial ecosystems in general [13, 14]. In Europe, intensified agricultural practices often include the removal of hedgerows to create large, homogenous, and intensively managed fields [15]. These practices have particular relevance for hedgehogs because hedgerows and field margins are important habitats for rural hedgehogs [16, 17]. In addition, these areas function as corridors connecting suitable habitats [18].

Habitat fragmentation by roads is famously a source of hedgehog mortality via roadkill [10, 19–21], but also has more subtle effects. Fragmentation reduces habitat connectivity, which is important for movement and dispersal and therefore has a direct effect on gene flow with consequences for fitness, adaptability and survival of local populations [22, 23]. Although habitat fragmentation is considered most pronounced in cities [24], where the dwindling green spaces can form crucial habitat networks [25], rural fragmentation remains a concern.

The small home range sizes and short nightly travel distances of hedgehogs mean that they are likely to be vulnerable to even small amounts of fragmentation. Although they can travel 2–3 km a night, adult hedgehog home ranges are ~20–30 ha for males and ~10 ha for females, expanding temporarily during the mating season [2]. Juveniles do not disperse far when reaching independence and leaving their natal nests [26] and adults appear to remain in the same area throughout their lives [1]. In addition, studies on relocated hedgehogs indicate that they do not disperse far even when released into a foreign [27] and unfavourable habitat [28].

In addition to habitat fragmentation, poisons, pollution and competition can affect hedgehog populations. The widespread application of molluscicides, insecticides and rodenticides can negatively influence hedgehog populations via secondary poisoning and elimination of prey items [29–32] and predation by, and inter-specific competition with, badgers (*Meles meles*) can be an important mortality source [9, 33–36]. Hedgehogs are increasingly associated with residential areas, possibly due to greater food availability around humans (including natural prey and anthropogenic sources), more suitable nest sites and a lower risk of predation by badgers [9, 28, 33–37].

## Genetic diversity of hedgehogs

Maintaining gene flow across fragmented landscapes is necessary to prevent the negative consequences of genetic drift, reduced genetic diversity, and inbreeding depression, which can result in less viable and less fertile offspring [38, 39]. The tools of conservation genetics, including genetic rescues, are useful for evaluating, conserving and managing genetically vulnerable, fragmented populations [40]. Research into the population genetics of hedgehogs is therefore useful for assessing conservation status.

Of particular concern in this context are the degrees of heterozygosity and inbreeding. Hedgehogs are promiscuous and have hetero-paternal superfecundation [41], which may reduce inbreeding, since a litter can consist of several half-siblings instead of full siblings [41]. This could be beneficial if mating does takes place between siblings in isolated populations. However, it is currently unknown whether hedgehogs are able to actively differentiate between kin and non-kin e.g. during the mating season. Nevertheless, it is clear that if a small, isolated population is severely inbred, and there is no distinction between kin and non-kin when choosing mates, the population will most likely become even more vulnerable due to the increased degree of inbreeding.

Previous genetic research on hedgehogs has been conducted using microsatellites [23, 41–53]. Bolfikova and Hulva (2012)[48] used population and landscape genetic approaches to describe the population structure and patterns of gene flow of *E*. *europaeus* and *E*. *roumanicus* in the central European contact zone between the two species. They found that a homogenous population of *E*. *europaeus*, had been divided by two large rivers in the Czech Republic (Vlatava and Elbe), into a subpopulation in the western part, and two subpopulations with a mosaic location pattern in the eastern part of the country. They found a significantly lower observed heterozygosity (H_O_) than expected heterozygosity (H_E_) in five of the nine microsatellite loci studied (n = 131), with a mean H_O_ of 0.695 and a mean H_E_ of 0.687, using the mitochondrial control region and nuclear microsatellites. In the UK, a study of 42 individuals in an isolated population of hedgehogs in the Regent’s Park, London (166 ha), showed a low genetic diversity, with a mean H_E_ of 0.197, and a mean H_O_ of 0.198 (n_loci_ = 6) [52]. Becher and Griffiths (1998)[43] detected a restriction of gene flow between eight small populations of hedgehogs in a 15 km^2^ fragmented landscape in Oxfordshire, UK and found a statistically significant genetic differentiation among the studied populations (n = 160, n_loci_ = 6) and a mean H_O_ of 0.7. According to the authors, these results indicated that the hedgehogs of Oxfordshire had a restricted dispersal which may have been caused by human-mediated barriers such as roads and train tracks in the landscape [43]. Braaker et al. (2017)[23] studied the habitat connectivity and spatial genetic structure of 147 hedgehogs residing in Zurich, Switzerland, with an area of 88 km^2^ (n_loci_ = 10). The population of hedgehogs in Zurich were divided into three genetic clusters, separated by two rivers and the major transportation axes. Genetic diversity measures were similar between the three clusters, and the F coefficients (F_IS_) were low. Mean H_E_ ranged between 0.569–0.627 for the three clusters, and the mean H_O_ ranged between 0.523–0.631 [23]. In summary, previous genetic studies on hedgehogs in Europe based on microsatellite techniques, have found a mean H_E_ ranging between 0.197 and 0.687.

### The Danish context

Hedgehogs have a long history in Denmark. Archaeological evidence from Mesolithic sites (Maglemosian cultures) confirms their presence in 7500 BC and other evidence suggests that they immigrated in the early Preboreal around 9550 BC [54]. Research into the glacial refugia and interglacial expansion of the European hedgehog showed three monophyletic clades [46]. The first from Italy northwards through Austria, Switzerland, Germany, the Netherlands, Scandinavia and Estonia. The second was only found in western Europe, from Spain northwards through France, the Netherlands and into the UK and Ireland. The third clade was restricted to Sicily. A single clade dominated in the Scandinavian hedgehogs [46].

Denmark consists of the large peninsula Jutland and several islands of varying sizes. The larger islands are connected by long bridges (0.75–17 km), which hedgehogs are unlikely to cross, isolating the local hedgehog populations in the different areas of Denmark. The total area of Denmark is 43,000 km^2^ [55], and 62% of this is arable land [56]. Altogether, Denmark has 74,728 km of roads and 1,737,000 cars for 5,800,000 people [57–59]. The number of cars in Denmark has increased with 22% since 2008 [58], and an increase in road traffic could likely influence the number of hedgehogs being killed by cars [60].

For this study we adapted and optimised the use of a second-generation genotyping technique, genotyping by sequencing (GBS) for the genetic analysis of European hedgehogs.

The main aims of this study were: i) To develop and test a set of SNPs which can be used for investigating the genetic structure and variability of the European hedgehog on a broader scale ii) to evaluate the patterns of the genetic diversity distribution in the Danish hedgehog populations, iii) to investigate potential anthropogenic effects on the genetic variability of the hedgehogs and iiii) to estimate the historical changes in their effective population size (N_e_) through genetic signatures.

## Materials and methods

The genetic samples were obtained as part of a nationwide citizen science project with the general aim of describing hedgehog ecology in Denmark. By use of local and national media and a project website, volunteers were encouraged to collect dead hedgehogs from May to December 2016. A total of 697 dead hedgehogs originating from all parts of Denmark were collected. The volunteers were instructed to record the date and location of the find and deliver the dead hedgehog to the nearest of 26 collection stations, distributed nationally. The hedgehog carcasses were stored locally at -20°C. Members of the research staff regularly transported the collected, dead hedgehogs to university laboratories, where they were thawed and necropsied from August 2016 to May 2018. During the necropsies, tissue samples from skin and muscle were obtained for the genetical analyses. The DNA samples were stored at -20°C.

Genetic samples from 178 individuals dispersed throughout Denmark were used in the study (Table 1, Supporting Information S1 Table and S2 Table [61]). The selection of samples was based on the quality of the extracted DNA from approximately 330 samples originating from all parts of Denmark. We chose 214 samples for further analyses. The later filtering of samples, described in the section “Filtering Raw Sequence Data, Mapping and SNP Calling”, reduced the sample size to 178 individuals.

**Table 1:** Distribution of genetic samples in the study.

Location	Abbreviation	Area in km^2^	Number of individuals
Jutland north of the Limfjord	JNL	5340	9
Jutland south of the Limfjord	JSL	23873	71
Funen	FN	3479	15
Zealand	Z	7031	51
Lolland and Falster	LFA	1797	18
Bornholm	BH	588	14
**Total:**			178

An overview of the distribution of the genetic samples in the study.

### Sample preparation

DNA was extracted from muscle and skin tissues (1–2 mg) using the DNeasy Blood and Tissue kit (QIAGEN, Germany) and subsequently digested with *Sau96I* (NEB) and ligated to adapters [62]. The ligated samples (50μL containing approximately 50 ng DNA pooled from 4 separate samples) were purified with AMPure XP beads (Beckman Coulter, USA) and amplified using the Phusion High-Fidelity PCR kit (Thermo Scientific, USA). The following PCR conditions were applied: 72°C in 5 min, 98°C in 30 sec, followed by 20 cycles of 98°C in 10 sec, 66°C in 30 sec and 72°C in 30 sec, with a final extension at 72°C for 5 min. Finally, the amplified barcoded DNA was purified with AMPure XP beads (Beckman Coulter, USA) and the DNA concentrations were determined by Qubit (Thermo Scientific, USA) and visualized on the TapeStation 2200 using a D1000 ScreenTape (Agilent, USA). Paired-end (2x151 bp) sequencing was performed on an Illumina HiSeq X platform by Admera Health (USA). The described method was previously tested in a pilot study using a few individuals from South Jutland by Rasmussen et al. (2019)[63].

### Barcoding analysis

The i7 barcodes of the dual-barcoded sequenced reads were demultiplexed using bcl2fastq2 version 1.0.0 (Illumina, USA) allowing zero mismatch. The i5 barcodes were demultiplexed using Fastq-multx version 1.02.772 (https://github.com/brwnj/fastq-multx) allowing one mismatch in order to remove the barcode sequences from the sequenced reads.

### Filtering raw sequence data, mapping and SNP calling

Adaptor quality trimming were accomplished by Trim Galore using default parameters (http://www.bioinformatics.babraham.ac.uk/projects/trim_galore). Burrows-Wheeler Aligner (BWA) was used to align the reads against the hedgehog (*Erinaceus europaeus*) using the EriEur2.26 reference genome. Only reads with a mapping quality of at least 30 were used for the further analysis. Variants were called using The Genome Analysis Tool Kit’s HaplotypeCaller, and joint genotyping was performed using GenotypeGVCFs. Initial filtering was performed using SelectVariants and filtered for SNPs, bi-allelic sites, and mapping quality > 30 [64]. Minor allele frequency (MAF) was estimated from the read coverage, and SNPs were filtered using a minimum of 1% MAF (average variant allele frequency < 0.99 and > 0.01). Finally, SNPs were filtered with a read coverage between 20 and 100 and a maximum number of missing data of 25%. Individuals with more than 25% missing data were not included in the analysis.

### Genetic variability and population structure

We assessed genetic diversity using four quantities: The percentage of polymorphic loci (P%), the F_IS_ coefficient (F_IS_), unbiased expected (uH_E_) and observed (H_O_) heterozygosity. These were all estimated using GENALEX v. 6.5 [65]. In addition, the individual level of heterozygosity (iH_O_) was calculated for every individual, plotted in a box plot, and tested for significant differences among locations with a one-way ANOVA. The pairwise comparisons among locations were conducted with a Tukey’s HSD test. The mean iH_O_´s within every population were ranked from the lowest to the highest value and plotted together in order to show the populations with the lowest and the highest levels of iH_O_.

Furthermore, the degree of genetic differentiation among the six populations was quantified with fixation index (F_ST_), and, for each population, a test for departure from Hardy-Weinberg equilibrium (HWE) was performed using GENALEX v. 6.5.

In addition to measuring genetic differentiation and variability, population genetic structure of the sampled individuals (n = 178) was assessed using a Bayesian clustering method, implemented with STRUCTURE v. 2.3, which clusters individuals into genetically distinct populations/groups (K) based on their allelic frequency at multiple loci and minimise deviations from HWE within groups [66]. To illustrate the population genetic structure of hedgehogs, 10 independent runs of K = 1–5 were carried out on the individuals with 10^6^ Markov chain Monte Carlo (MCMC) iterations and 10^5^ burn-in period on the basis of independent allele frequencies and admixture ancestry model. Additionally, we ran STRUCTURE of K = 1–7 separately for every population to test if further substructuring could be detected within the particular populations. The accurate number of populations (K) was determined according to the ΔK formula [66] using the program STRUCTURE HARVESTER [67]. In addition to this Bayesian clustering approach, a multivariate ordination of individual genotypes was obtained by principal component analysis (PCA) using the software GENODIVE [68] and plotted with the software PAST version 1.90 [69].

### Assessment of the demographic history

Tests for recent population declines or expansions in the population size were performed for every population with the program BOTTLENECK v. 1.2, after 15,000 iterations assuming an infinite allele model (IAM) according to a deficiency of the rare alleles and an excess of the heterozygosity. The model assumes that a population (large size) is in mutation-drift equilibrium and that as a consequence of a severe restriction in size the loss of alleles (especially the rare ones) occurs at a higher pace that the loss of the genic heterozygosity. Consequently, a deficiency in the total number of alleles is detected when compared to the number of expected alleles in a large population with the same observed heterozygosity. The deficiency in allele number will only be apparent until the population reaches the mutation-drift equilibrium again [70, 71].

### Estimation of potential anthropogenic effects on genetic variability of hedgehogs

To investigate whether the differences in genetic variability found between the hedgehog populations were associated with human population density in their area, road density and farmland density of the areas from which the studied hedgehogs derived, we fitted linear models in R [72]. The response variables were iH_O_, uH_E_, F_IS_ and % polymorphic loci (%P) and the explanatory variables were human population density per km^2^ [73, 74], farmland per km^2^ [75], and kilometres of roads per km^2^ [58] (The dataset used is available in Supporting Information S3 Table [61]).

For each of the four response variables (iH_O_, uH_E_, F_IS_ and %P) we first fitted models with a single explanatory variable at a time (i.e. three models for each of the three response variables), followed by linear models with two explanatory variables at a time. Then we fitted models that included all three explanatory variables as main effects. Lastly, we prepared models that included two explanatory variables at a time (e.g. road density and farmland density) and the interactions between them. We tested the significance of the interaction term by comparing models that included the interaction term to those that did not include the interaction term, using an ANOVA test.

### Research ethics

Ethical approval was not required for this research, because the hedgehogs used in the study had already died of natural causes either in the wild or in care at a hedgehog rehabilitation centre. Additionally, the volunteers deciding to assist in the collection of dead hedgehogs did so of their own free will and were instructed on how to keep a high hygienic standard and prioritise traffic safety when collecting the dead hedgehogs through our project website.

## Results

### Genotypes

Using the *Sau96I* restriction enzyme we were able to recover a total of 2.4 million high-quality SNPs. Filtering for MAF > 1% estimated from the read coverage, a maximum of 25% missing data and a read coverage ranging from 20 to 100, resulted in 2902 applicable SNPs. Individuals containing less than 75% of the selected SNPs were not included in the analysis and this resulted in 178 individuals used for studying the genetic variability (the GENEPOP dataset is available in Supporting Information S2 Table [61]).

**Fig 1:**
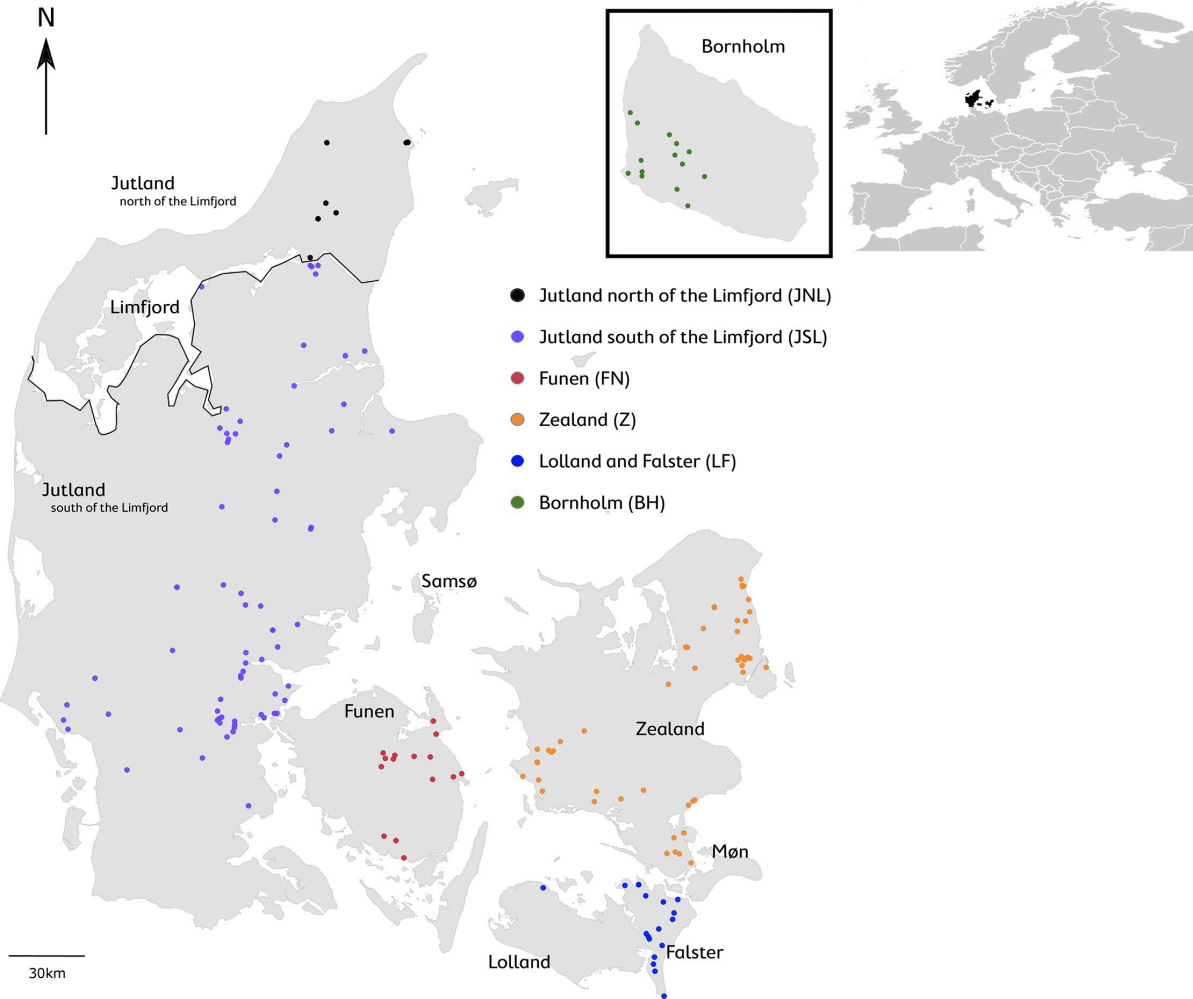
Geographical overview of the samples. Map of Denmark indicating the locations of the 178 hedgehogs used in the study.

The geographical sampling locations reported by the volunteers are shown in Fig 1, representing Jutland north of the Limfjord (JNL), Jutland south of the Limfjord (JSL), Funen (FN), Zealand (Z), Lolland and Falster (LFA) and Bornholm (BH). The location data may have varying levels of accuracy, because volunteers provided this data without verification.

### Genetic variability and structure

**Fig 2:**
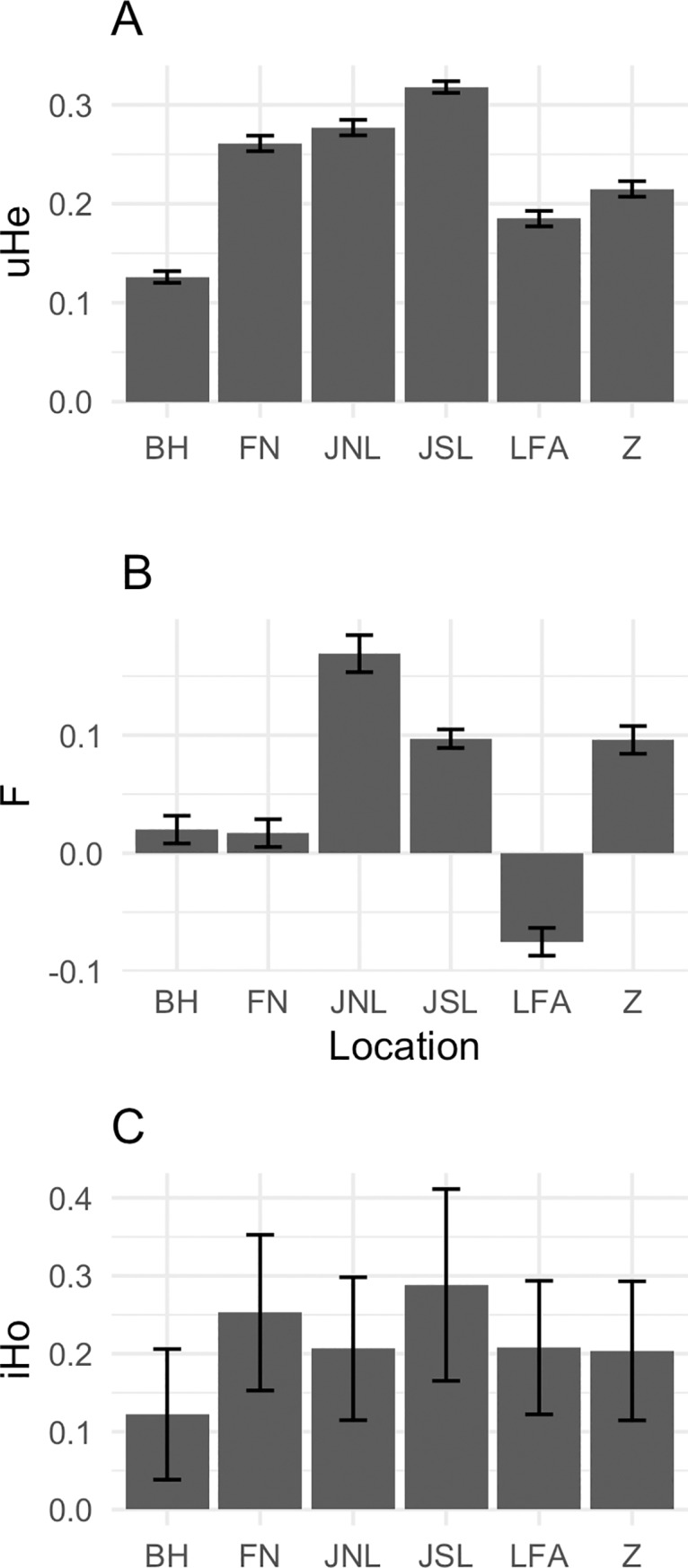
Plots of genetic heterozygosity and location. Measures of (A) unbiased expected heterozygosity (uH_E_) and (B) F coefficient (F_IS_) and (C) individual observed heterozygosity (iH_O_), vary with location. Height of the bars indicate the estimated value and error bars represent the 95% confidence intervals of these estimates. Locations are as follows: BH = Bornholm, FN = Funen, JNL = Jutland north of the Limfjord, JSL = Jutland south of the Limfjord, LFA = Lolland and Falster and Z = Zealand.

We found that the unbiased expected heterozygosity (uH_E_), mean individual heterozygosity iH_O_ and the F coefficient (F_IS_), varied with location (Fig 2). uH_E_ and iH_O_ showed marked variation among regions, with the lowest value reported on Bornholm (BH) and the highest in Jutland south of the Limfjord (JSL). The measure of uH_E_ was very similar in Funen (FN) and Jutland north of the Limfjord (JNL). Mean iH_O_ values from Lolland and Falster (LFA), Jutland north of the Limfjord (JNL) and Zealand (Z) were almost identical. Of the three statistically significant and positive F_IS_ values (FN, JSL, Z) the levels were similar between Jutland south of the Limfjord (JSL) and Zealand (Z), but markedly lower on Funen (Fig 2, Table 2).

**Table 2:** Table of genetic heterozygosity.

Locations	Abbreviations	Area km^2^		uH_E_	F_IS_	H_O_	iH_O_	%P	HWE test
Jutland north of the Limfjord	JNL	5340	Mean	0.277	0.169	0.212	0.206	72.72	NS
			SE	0.004	0.008	0.004	0.016		
Jutland south of the Limfjord	JSL	23873	Mean	0.318	0.097	0.293	0.288	94.79	***
			SE	0.003	0.004	0.004	0.008		
Funen	FN	3479	Mean	0.261	0.017	0.253	0.253	79.08	***
			SE	0.004	0.006	0.004	0.013		
Zealand	Z	7031	Mean	0.215	0.096	0.206	0.204	87.08	***
			SE	0.004	0.006	0.004	0.006		
Lolland and Falster	LFA	1797	Mean	0.185	-0.075	0.208	0.208	67.73	NS
			SE	0.004	0.006	0.005	0.010		
Bornholm	BH	588	Mean	0.126	0.02	0.124	0.122	54.44	NS
			SE	0.003	0.006	0.004	0.011		

Table presenting the percent of polymorphic loci (P%), F coefficient (F_IS_), unbiased expected (uH_E_), observed (H_O_) and individual observed (iH_O_) heterozygosity and a test for Hardy-Weinberg equilibrium (HWE) for the six hedgehog populations studied.

*** = p < 0.0001. Supporting Information S3 Table [61]contains further information on regional differences.

The genetic polymorphism increased according to the size of the regions, ranging from 54.44% in BH to 94.79% in JSL (Table 2).

The uH_E_ ranged from uH_E_ = 0.126 in BH to uH_E_ = 0.318 in JSL whereas, the H_O_ ranged from H_O_ = 0.124 in BH to H_O_ = 0.293 in JSL (see Table 2).

We detected a significant deviation for HWE in JSL, FN and Z and in all cases the deviations were due to a deficiency of heterozygotes which is also reflected by the positive F_IS_ values which ranged from -0.075 in LFA to 0.17 in JNL (Table 2).

A box plot of the individual heterozygosity iH_O_ for every individual is presented in the Supporting Information S1 Fig [61]. The one-way ANOVA conducted for testing significance of the mean iH_O_ among the six populations investigated was highly significant: F _5,172_ = 31.92, p < 0.0001). The Tukey’s test found several significant differences of the mean iH_O_ between populations (Supporting Information S4 Table [61]). The JSL population had significantly higher iH_O_ compared to all the remaining populations with the exception of FN population. In addition, BH had a significantly lower mean iHo than all the other populations. Lastly, FN has significantly higher iH_O_ than Z. The plots of the iH_O_ ranked in ascending order is showing that the populations with the lowest iH_O_ are BH, followed by JSL which is however quickly increase in iH_O_ (indicating that few individuals have low values), followed by Z, JNL, LFA and finally FN which showed the highest starting levels of iH_O_ (Supporting Information S1 Fig and S2 Fig [61]).

We found that all the pairwise fixation index (F_ST_) values between populations were highly significant (p < 0.001) with a F_ST_ range from 0.034 between JNL and JSL and F_ST_ = 0.321 between JNL and BH (see Table 3), indicating that the six populations of hedgehogs tested are genetically different.

**Table 3:** Pairwise F_ST_ values matrix.

JNL	JSL	FN	Z	LFA	BH	
						JNL
0.034						JSL
0.146	0.108					FN
0.201	0.159	0.046				Z
0.237	0.191	0.063	0.030			LFA
0.321	0.268	0.192	0.182	0.204		BH

Pairwise F_ST_ values matrix. All the F_ST_ values were highly significant (p< 0.001). Locations: JNL = Jutland north of the Limfjord, JSL = Jutland south of the Limfjord, FN = Funen, Z = Zealand, LFA = Lolland and Falster and BH = Bornholm.

The Bayesian clustering of the genotyped data assigned the highest posterior probability: Estimated Ln Prob of Data = -144614.8, Variance of ln likelihood = 1958.7 for K = 3 (the plot of the Ln Prob of Data versus K is available in the Supporting Information S3 Fig [61]). We found that the three clusters included: 1) Jutland north of the Limfjord (JNL) and Jutland south of the Limfjord (JSL); 2) Funen (FN), Zealand (Z), Lolland and Falster (LFA); 3) Bornholm (BH). We did not observe further evidence of subtructuring within populations when testing for K = 4 and K = 5. These findings were also confirmed by the lack of further substructuring, when testing for K = 1–7 for each population analysed separately (Supporting Information S4 Fig [61]).

**Fig 3:**
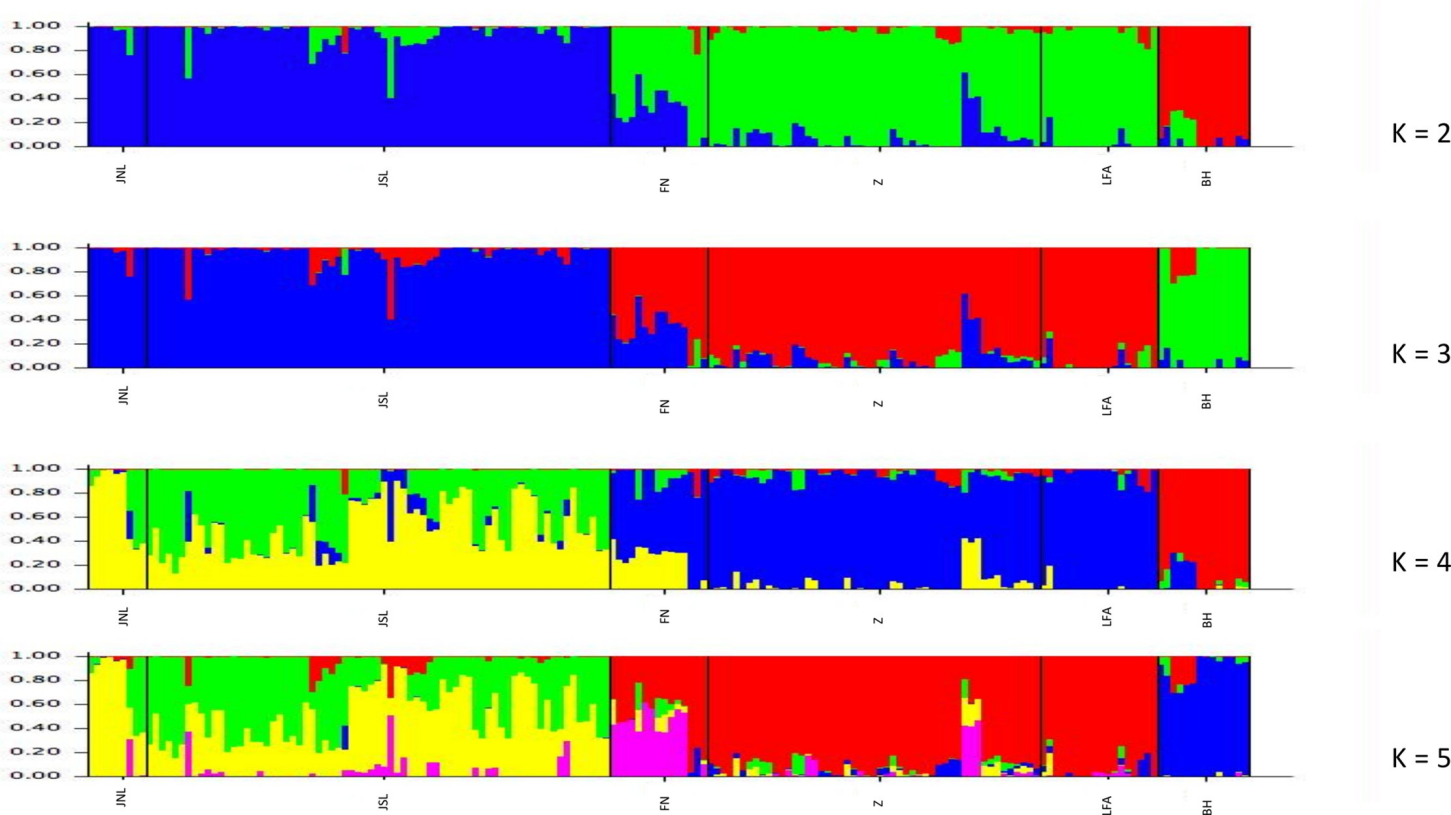
STRUCTURE analysis plots. Plots of the STRUCTURE analysis for K = 2–5 for all the six detected populations: Jutland north of the Limfjord (JNL), Jutland south of the Limfjord (JSL), Funen (FN), Zealand (Z), Lolland and Falster (LFA) and Bornholm (BH).

The PCA (Supporting Information S5 Fig [61]) was concordant with the clustering of STRUCTURE (Fig 3), the PC1 (Eigenvalues 19.54; variance explained; 79.24%) and PC2 (Eigenvalues 5.12; variance explained; 20.76%) and the convex hulls kept the three clusters well separated (Supporting Information S5 Fig [61]).

### Demographic changes

We found no signs of recent increase or decline in the population in the six populations tested by use of the infinite allele model (IAM).

### Estimation of potential anthropogenic effects on genetic variability of hedgehogs

Testing for effects of farmland density, population density and road density in the particular areas from where the hedgehogs derived on the genetic variability observed in the study, we found no statistically significant associations between iH_O_, uH_E_ and % polymorphic loci (%P) for each of the six populations and farmland density, road density or population density, fitted as single main effects (Table 4, the dataset used is available in Supporting Information S3 Table [61]). Models representing two or three explanatory variables at a time as main effects, with iH_O_, uH_E_ or %P as response variables, showed no significant associations. We furthermore tested for a significant interactive effect between the different explanatory variables by comparing models with and without the interactions, but again found no significant relationship. Additionally, the explanatory variables road density and population density had no statistically significant effect on F_IS_. However, we found a statistically significant association between F_IS_ and farmland density (Table 4). F_IS_ decreases with increased farmland density (with a factor 1.0315). Models fitted with farmland density and road density, as well as all three explanatory variables at a time, as main effects, also showed significant associations between farmland density and F_IS_.

**Table 4:** Statistical results from the estimation of potential anthropogenic effects on genetic variability of hedgehogs.

Explanatory variables	iH_O_	uHE	F_IS_	%P
Road density	F[1,4] = 0.5045, p = 0.517	F[1,4] = 0.935, p = 0.388	F[1,4] = 0.019, p = 0.898	F[1,4] = 0.004, p = 0.95
Farmland density	F[1,4] = 0.00006, p = 0.994	F[1,4] = 0.405, p = 0.559	F[1,4] = 9.603, p = 0.036*	F[1,4] = 0.456, p = 0.537
Population density	F[1,4] = 0.011, p = 0.921	F[1,4] = 0.003, p = 0.958	F[1,4] = 0.356, p = 0.583	F[1,4] = 1.184, p = 0.338
Road density + Farmland density	F = 0.381, p = 0.581 F = 0.019, p = 0.898	F = 0.89, p = 0.415 F = 0.81, p = 0.434	F = 0.063, p = 0.394 F = 10.491, p = 0.048*	F = 0.006, p = 0.944 F = 0.342, p = 0.600
Population density + Road density	F = 0.014, p = 0.913 F = 2.161, p = 0.238	F = 0.006, p = 0.943 F = 4.808, p = 0.116	F = 0.375, p = 0.584 F = 1.212, p = 0.351	F = 11.700, p = 0.283 F = 2.746, p = 0.196
Farmland density + Population density	F = 0.001, p = 0.972 F = 0.008, p = 0.933	F = 0.321, p = 0.610 F = 0.003, p = 0.963	F = 6.375, p = 0.086 F = 0.835, p = 0.4283	F = 0.414, p = 0.566 F = 0.633, p = 0.484
Road density + Farmland density + Population density	F = 0.389, p = 0.597 F = 0.020, p = 0.901 F = 1.063, p = 0.411	F = 1.209, p = 0.386 F = 1.100, p = 0.404 F = 2.073, p = 0.287	F = 0.124, p = 0.758 F = 20.851, p = 0.045* F = 3.962, p = 0.185	F = 1.922, p = 0.300 F = 0.100, p = 0.782 F = 1.190, p = 0.389
Road density: Farmland density	ANOVA, F = 0.305, p = 0.636	ANOVA, F = 0.348, p = 0.615	ANOVA, F = 0.030, p = 0.878	ANOVA, F = 0.106, p = 0.927
Road density: Population density	ANOVA, F = 1.416, p = 0.356	ANOVA, F = 10.756, p = 0.082	ANOVA, F = 0.377, p = 0.602	ANOVA, F = 0.842, p = 0.456
Farmland density: Population density	ANOVA, F = 1.187, p = 0.708	ANOVA, F = 0.151, p = 0.735	ANOVA, F = 0.018, p = 0.906	ANOVA, F = 0.017, p = 0.909

Results from the analyses of potential anthropogenic effects on the genetic variability of hedgehogs, based on linear models including one or more of the three explanatory variables (road density, farmland density or population density) tested against the four response variables iH_O_, uH_E_, F_IS_ and %P. The colon (:) indicates models with an interaction term between the explanatory variables, which were then compared to models that did not include the interaction term, using an ANOVA test. Significant p-values are marked with *.

## Discussion

### Genetic variability and structure

Previous studies have compared the applicability of microsatellite and SNP as linkage mapping markers [76, 77], mentioning that the advantage of microsatellite markers are their highly polymorphic characteristics compared to the biallelic SNPs. However, the high polymorphism in the microsatellite markers may cause relatively high genotyping errors. The advantage of SNPs is their greater density in the sample, providing a higher information content, even though they provide less information per locus. Hence, their lower variability causes a need for an increased number of markers compared to the microsatellite approach [76, 77].

Preceding genetic studies on hedgehogs in Europe based on microsatellite techniques, have found a mean H_E_ ranging between 0.197 and 0.6872. A calculated mean H_E_ of 0.197 and a mean H_O_ of 0.198 (n = 42) was reported in an isolated population of hedgehogs in Regent’s Park, London [52], while a mean H_O_ of 0.7 (n = 160) was determined in a study from Oxfordshire, UK [43]. A mean H_E_ of 0.6872, a mean H_O_ of 0.695 and a mean F_IS_ of 0.0686 was determined in hedgehogs from the Czech and Slovak Republics (n = 131) [48]. Mean H_E_ ranged between 0.569–0.627 (n = 147) and the mean F_IS_ ranged between -0.006–0.070 for three genetic clusters in Zurich, Switzerland [23]. In our research based on the GBS technique, we found a mean uH_E_ varying between 0.126–0.318 and a mean H_O_ varying between 0.124–0.293 for the Danish hedgehogs studied (Table 2). As Denmark is a relatively small area compared to e.g. continental Europe, with an isolated island structure, and thereby has a reduced gene flow, a lower heterozygosity would be expected for the Danish hedgehogs. The peninsula of Jutland south of the Limfjord is connected to Germany, which could potentially have caused a higher gene flow, but we still found a positive F_IS_ coefficient and low heterozygosity in this population (H_O_ = 0.293, uH_E_ = 0.318, F_IS_ = 0.097). However, individuals from Jutland south of the Limfjord did in fact have the highest measures of heterozygosity (H_O_ and uH_E_) of all the areas tested in the present study (Fig 2, Table 2). Furthermore, in comparison to using SNPs with only two possible alleles, the microsatellite approach in the previous studies may also overestimate heterozygosity due to the polymorphic nature of the microsatellites, causing a direct comparison between the results of the two approaches to be scientifically unsound [78].

Both the STRUCTURE analysis (Fig 3 and Supporting Information S4 Fig [61]) and PCA plot (Supporting Information S5 Fig [61]) in the present study were concordant. It is quite evident that the population in Jutland (JNL, JSL) forms one single cluster whereas all the remaining islands tested, apart from BH, form the second cluster. Lastly, the STRUCTURE analysis plots (Fig 3) are clearly indicating an admixed structure on the island of FN, as also supported by findings in the PCA plot (Supporting Information S5 Fig [61]). The plot of the iH_O_ values for each of the six populations ranked from the lowest to the highest values within each population (Supporting Information S2 Fig [61]) are also showing that the iH_O_ for FN has the steepest cumulative curve, indicating a higher heterogeneity among individuals for the iH_O_ values, which can be due to further substructuring or the presence of inbreed individuals. Despite the evidence for an admixed structure on the island of FN, the log-likelihood plot for K > 1 for the island of FN failed to find significantly higher log-likelihood for K = 2 compared to the log-likelihood of K = 1, rejecting the evidence for genetic substructuring. One reason could be the small sample size of the hedgehogs analysed on the FN island. It is noteworthy that the iH_O_ values for the other populations show heterogeneity among individuals both in terms of lowest iH_O_ values and in terms of slopes, which indicate the presence of further substructuring and/or inbreeding (Supporting Information S2 Fig [61]).

Lastly, the island of BH is separated from the second cluster (FN, Z, LFA) even if partially overlapping with it, and is clearly separated from the first cluster (JSL, JNL). The island of Bornholm, which was previously connected to the continent around northern Germany, became an island in late Preboreal approximately 8000 BC [79]. The rest of Denmark was part of a large continent connected with current areas such as UK and southern Sweden [79], but was transformed into islands and the peninsula of Jutland around 6000–6500 BC [54], when the North American ice shield melted and made the oceans rise. At that point in time, Denmark was surrounded by the Littorina Sea, Lolland and Falster was still connected, and the area south and north of the Limfjord and Djursland as well as northern Zealand consisted of archipelagos [79]. It was not until around 6000 BC that the current geography of Denmark was shaped. Previous research indicates that Lolland and Falster was divided by the sound Guldborgsund around 4000 BC [80], but may have been periodically connected up until 1000 AD [81], which could explain the non-significant difference of F_ST_ found between the two islands before we decided to merge them into one population. Jutland north and south of the Limfjord have regularly been connected by different isthmuses closing off the western entrance to the Limfjord from the North Sea from around 1200 AD. In 1863 it was decided to artificially maintain an opening between the Limfjord and the North Sea [82]. The periodical connection between Jutland north and south of the Limfjord could have influenced the genetic cluster found between those two populations of hedgehogs.

The F_ST_ values were all highly significant, however, because several of the populations investigated are not in HWE the F_ST_ values should be interpreted with caution, as one of the assumptions for a correct estimate test is that the populations which are compared, are panmictic.

JNL, LFA and BH had a low genetic variability (Table 2). However, these three populations did have considerably smaller sample sizes than JSL and Z, which could have affected the results (as the small sample size increase the possibility of committing an error type II; false negative). As an example, JNL had an F coefficient (F_IS_) of 16.9%, but the HWE test was still negative. This could be due to the small sample size or the large standard errors of uH_E_ and H_O_.

JSL, FN and Z showed a low genetic variability (Table 2), and as can be seen from the STRUCTURE plot for K = 3 (Fig 3), there is evidence for further substructuring and/or non- panmictic populations. The STRUCTURE analysis failed to find further substructuring. The significant deviation from HWE observed in three of the populations investigated could reinforce the hypothesis of barriers to gene-flow such as habitat fragmentation, as the deviation from HWE could be due to further substructuring of the populations investigated (Wahlund effect) which produces a heterozygosity deficiency due to the lack of panmixia, as seen in hedgehog populations, where competition for the favor of females often occur. Additionally, female hedgehogs are selective of their mates, which often results in courtship without mating [83].

An additional possible factor, which could have contributed to the deviations from HWE, is the effect of humans relocating hedgehogs into foreign habitats. During the past 20–30 years, the rehabilitation of sick, orphaned and injured wild hedgehogs has become an established practice in many western European countries. Denmark has a number of working hedgehog rehabilitation centres, where volunteers care for the hedgehogs with the purpose of releasing the surviving individuals back into the wild. In 2013 the Danish Nature Agency prepared guidelines for the care of wildlife, instructing rehabilitators to refrain from moving mammals over water, and ensuring that rehabilitated wildlife would be released back into their original habitat [84]. Injured wildlife from e.g. Jutland north of the Limfjord, should therefore only be admitted to a wildlife rehabilitation centre north of the Limfjord and be released into the original habitat. Only recently, in 2019, have the Danish authorities established legal frameworks and monitoring programs for the practice of wildlife rehabilitation. The practice before 2013 was to transport wildlife in need of care to the nearest rehabilitation centre, which could be situated far away from the original habitat. Often the animals would be released near the rehabilitation centre. There are previous examples of hedgehogs from e.g. the small island of Ærø being cared for in the northern part of Zealand 200 km away, crossing the seas of the South Funen Archipelago and the Great Belt, most likely because people had brought the sick hedgehog with them when returning from vacation. Furthermore, Kristiansson (1981) [85] showed that intentional introductions of hedgehogs could have influenced the distribution of hedgehogs in Sweden, Norway and Finland. The anthropogenic effects on hedgehog genetics in Denmark could largely be explained by the translocation of hedgehogs between different parts of Denmark.

One drawback to our citizen science sample collection methods was that we did not have precise and reliable records of the geographical location of the samples. This had consequences for the analyses we could carry out because analyses such as Isolation by Distance (IBD) and the Mantel test are very sensitive to even small deviations from the precise location point. We therefore refrain from interpreting the conducted Mantel and IBD tests. Future collection of genetic samples should endeavor to collect precise locations for the samples.

### Effective population size and population bottleneck and expansion

The low level of genetic variability can be explained by inbreeding, genetic substructure and extremely low N_e_ or a combination between these factors which could be caused by habitat fragmentation and/or the large amount of farmland in Denmark. As intensified agricultural practises increase, arable land is gradually becoming a less suitable habitat for hedgehogs. The decline in the hedgehog population of the UK has even been found to be more severe in rural areas than urban [5]. Two-thirds of the area of Denmark is arable land [56]. In comparison, the share of total area by type and land cover in percentage of the EU countries show that the amount of cropland is particularly high in Denmark (50.2%) compared to e.g. Austria (15.3%), United Kingdom (19.7%), Slovakia (26.6%) and the Czech Republic (32%) [86], where previous studies on hedgehog genetics found remarkably high genetic variability using microsatellites [23, 43, 48]. However, as the approach to determine the genetic heterozygosity in the present study (the GBS technique using SNPs) differs from the previous research, the variation of genetic variability between the studies should be interpreted with caution and the results should not be compared directly.

We found an effect of farmland density on the F coefficients (F_IS_) in our study, but the effect was surprisingly that F_IS_ decreased with increasing farmland density. This may be due to the tendency for a lower degree of road-associated fragmentation in farmland areas and less traffic in general. Or perhaps because the hedgehogs, which are able to survive in this habitat type, can move more freely in their search for mates, because the limitations of movement primarily present in urban areas such as buildings, fences and traffic-laden roads, are less pronounced in farmland areas. Further research is needed to understand this effect.

Habitat fragmentation can cause founder effects [87] and, because hedgehogs have relatively small home ranges and are not dispersing far from their birthplace, they are vulnerable to habitat fragmentation and barriers of movement in general. Consequently, a conservation campaign has been established in the UK, where citizens are encouraged to make holes in their fences to increase garden connectivity for the hedgehogs. Roads as barriers causing habitat fragmentation are also a challenge for hedgehogs, as they are often killed in traffic when crossing roads, especially during the mating season, where males increase their home ranges in search for mates [83]. We tested the possible effects of road density, as a measure of habitat fragmentation in the area from which the hedgehogs derived, on the genetic variability found. We failed to find an association between road density and iH_O_, F_IS_, %P and uH_E_.

We also tested whether the human population density per area for each population of hedgehogs studied, had an effect on iH_O_, F_IS_, %P and uH_E_. JSL, FN and Z, where we found inbreeding and/or subpopulations, had the highest number of citizens per area. This measure may indicate that hedgehogs in these areas are under stronger influence of anthropogenic effects caused by factors such as more cars and traffic and more construction sites replacing hedgehog habitats. However, we failed to find an association between population density and iH_O_, F_IS_, %P and uH_E_ in the hedgehogs studied. However, the tests for effects of population density, farmland density and road density were based on a small sample size (n = 6 populations), which has likely influenced the statistical power of the tests.

The software BOTTLENECK 1.2. failed to detect signs of population bottlenecks or increase in population size. However, the software only detects decreases or increases in population size, which has occurred recently (within 0.2 N_e_ to 0.4 N_e_ generations). Therefore, we cannot reject the possibility that the population is declining and or have declined drastically before the scope of 0.2 to 0.4 N_e_ generations.

## Conclusions

We adapted the GBS technique with the application of 2902 SNPs per individual to investigate the genetic structure and variability of the European hedgehog on a broader scale. By applying the technique to samples from 178 Danish hedgehogs, we found a low genetic variability (H_O_ = 0.124–0.293). We detected differences between the mean iH_O_ in the populations, which indicate some degree of inbreeding and fragmentation. The 178 Danish hedgehogs tested could be divided into six geographically distinct populations based on the Danish island structure and hence isolation by water. This division was furthermore confirmed by the pairwise fixation index (F_ST_). The STRUCTURE analysis determined that the six populations were distributed inside three genetic clusters. Investigating the potential anthropogenic effects on the genetic variability of the hedgehogs, we discovered that the inbreeding coefficient (F_IS_) decreased with increasing farmland density, but we found no evidence for an effect of human population or road density. We found evidence for genetic substructure and low effective population size for all populations, which are all consequences of habitat fragmentation. Furthermore, no signs of a recent population bottleneck or population increase or decline were detected.

There is a valuable potential for further analyses such as individual-based landscape genomic studies testing for the effect of landscape attributes on the genetic diversity and connectivity, if precise location data and environmental parameters are provided, enabling the correlation of genetic parameters like uH_E_, H_O_, %P and F_IS_. Given the lack of knowledge on the population status of Danish hedgehogs, we believe that future research on hedgehog genetics should focus on the effects of low individual genetic heterozygosity to determine the impact of inbreeding on individual fitness including indicators such as dental health, parasitic load, microbiomes, toxicology and prevalence of cancer.

## Supporting information

S1 Table Overview of individuals. Overview of the individuals used in the genetic sampling.
(TIFF)

S2 Table Dataset from GENEPOP. A presentation of the dataset from GENEPOP.
(TIFF)

S3 Table Data set for the linear models testing for anthropogenic effects on hedgehog heterozygosity. The data applied in the analyses with linear models to investigate anthropogenic effects on hedgehog heterozygosity. Area km^2^ describes the area of the regions measured in km^2^ [74]. Population/Area is a measure of the human population density per km^2^ in the regions [73, 74]. Farm/Area is a farmland per km^2^ in the regions [75], and Roads/Area indicates km of roads per km^2^ in the regions [58].
(TIFF)

S4 Table Tukey’s test matrix. Tukey’s test matrix for testing pairwise significant differences of the mean iH_O_ between the six populations: Jutland north of the Limfjord (JNL), Jutland south of the Limfjord (JSL), Funen (FN), Zealand (Z), Lolland and Falster (LFA) and Bornholm (BH).
(TIFF)

S1 Fig Box plot of the individual heterozygosity (iH_O_). Box plot of the individual heterozygosity (iH_O_) estimated for the six populations: Jutland north of the Limfjord (JNL), Jutland south of the Limfjord (JSL), Funen (FN), Zealand (Z), Lolland and Falster (LFA) and Bornholm (BH).
(TIFF)

S2 Fig Plot of the iH_O_ values for the six populations. Plot of the iH_O_ values for each of the six populations ranked from the lowest to the highest values within each population.
(TIFF)

S3 Fig Likelihood plot of STRUCTURE results. Likelihood plot of STRUCTURE results. Ln P(D) is the mean likelihood of K, the number of simulated clusters. The most likely K is that where ln P(D) is maximized.
(TIFF)

S4 Fig Likelihood plot of STRUCTURE results. The figures are representing the Likelihood plots of results for each of the populations run separately in STRUCTURE (JNL, JSL, FN, Z, LFA, BH). Ln P(D) is the mean likelihood of K, the number of simulated clusters. The most likely K is that where ln P(D) is maximized. Jutland north of the Limfjord (JNL), Jutland south of the Limfjord (JSL), Funen (FN), Zealand (Z), Lolland and Falster (LFA) and Bornholm (BH).
(TIFF)

S5 Fig Principal component analysis. Principal Component Analysis of the PC1 (Eigenvalues 19.54; variance explained; 79.24%) and PC2 (Eigenvalues 5.12; variance explained; 20.76%) and the convex hulls. The following colours are indicating the six discovered populations: JNL: Jutland north of the Limfjord (Black); JSL: Jutland south of the Limfjord (Purple); FN: Funen (Red); Z: Zealand (Orange); LFA: Lolland and Falster (Blue); BH: Bornholm (Green).
(TIFF)
