# Supplementary figures and images for: Genetic structure of the European hedgehog (Erinaceus europaeus) in Denmark
Source: PLoS One. 2020 Jan 17;15(1):e0227205. doi: 10.1371/journal.pone.0227205 (PMC6968871; doi:10.1371/journal.pone.0227205)

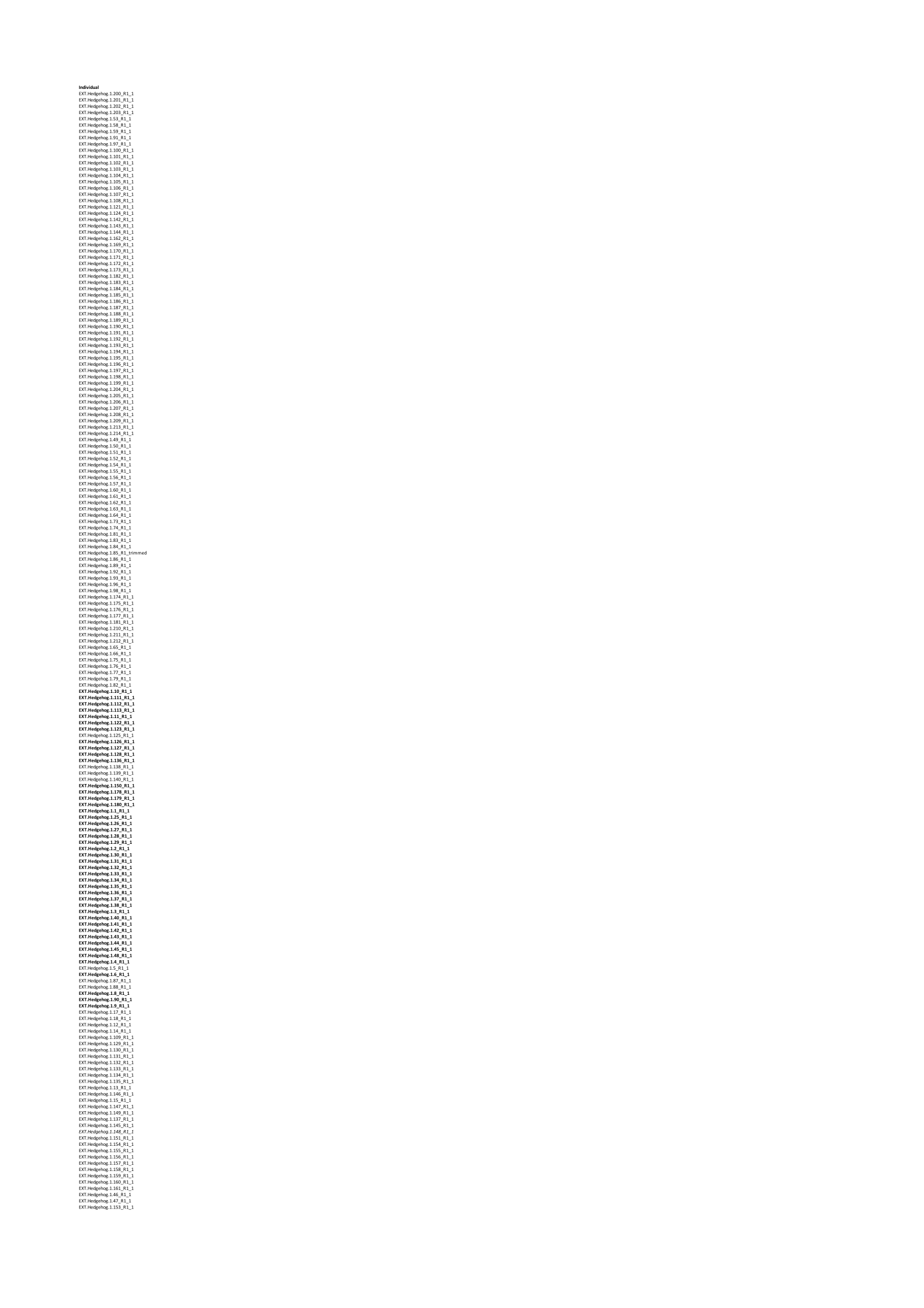

Supplement: S1 Table — Overview of the individuals used in the genetic sampling. (TIFF) [file pone.0227205.s001.tiff]

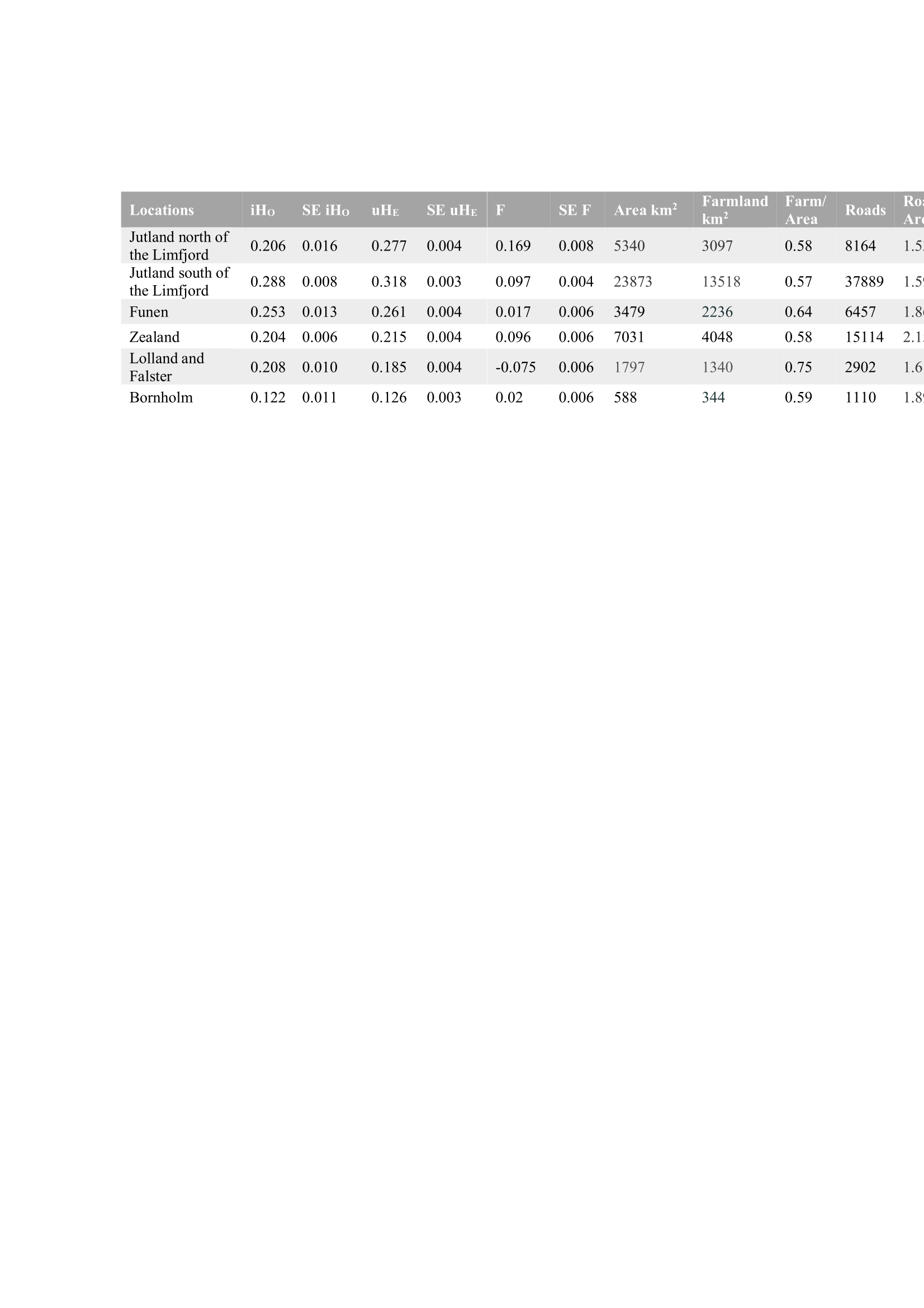

Supplement: S3 Table — The data applied in the analyses with linear models to investigate anthropogenic effects on hedgehog heterozygosity. Area km2 describes the area of the regions measured in km2 [74]. Population/Area is a measure of the human population density per km2 in the regions [73, 74]. Farm/Area is a farmland per km2 in the regions [75], and Roads/Area indicates km of roads per km2 in the regions [58]. (TIFF) [file pone.0227205.s003.tiff]

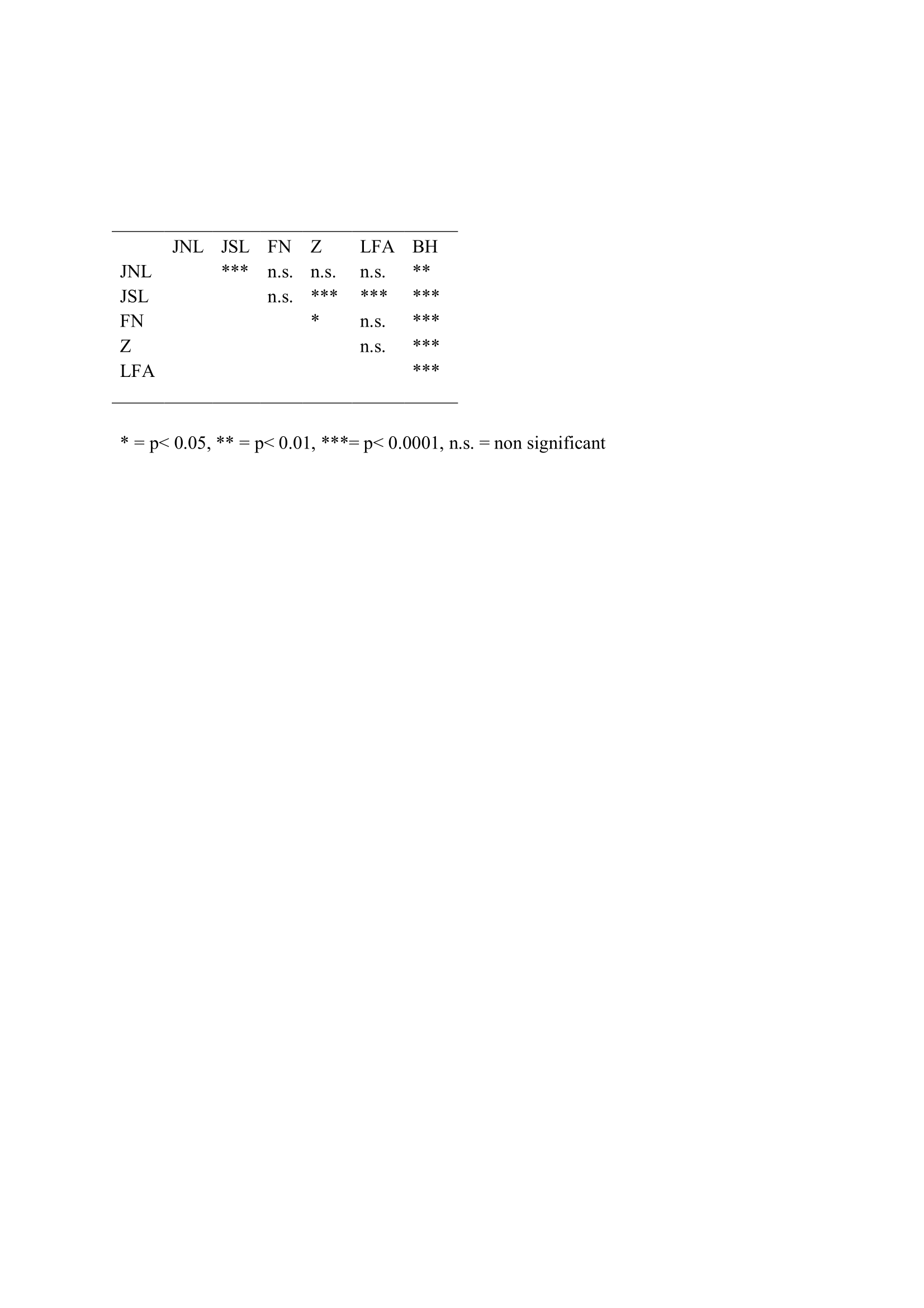

Supplement: S4 Table — Tukey’s test matrix for testing pairwise significant differences of the mean iHO between the six populations: Jutland north of the Limfjord (JNL), Jutland south of the Limfjord (JSL), Funen (FN), Zealand (Z), Lolland and Falster (LFA) and Bornholm (BH). (TIFF) [file pone.0227205.s004.tiff]

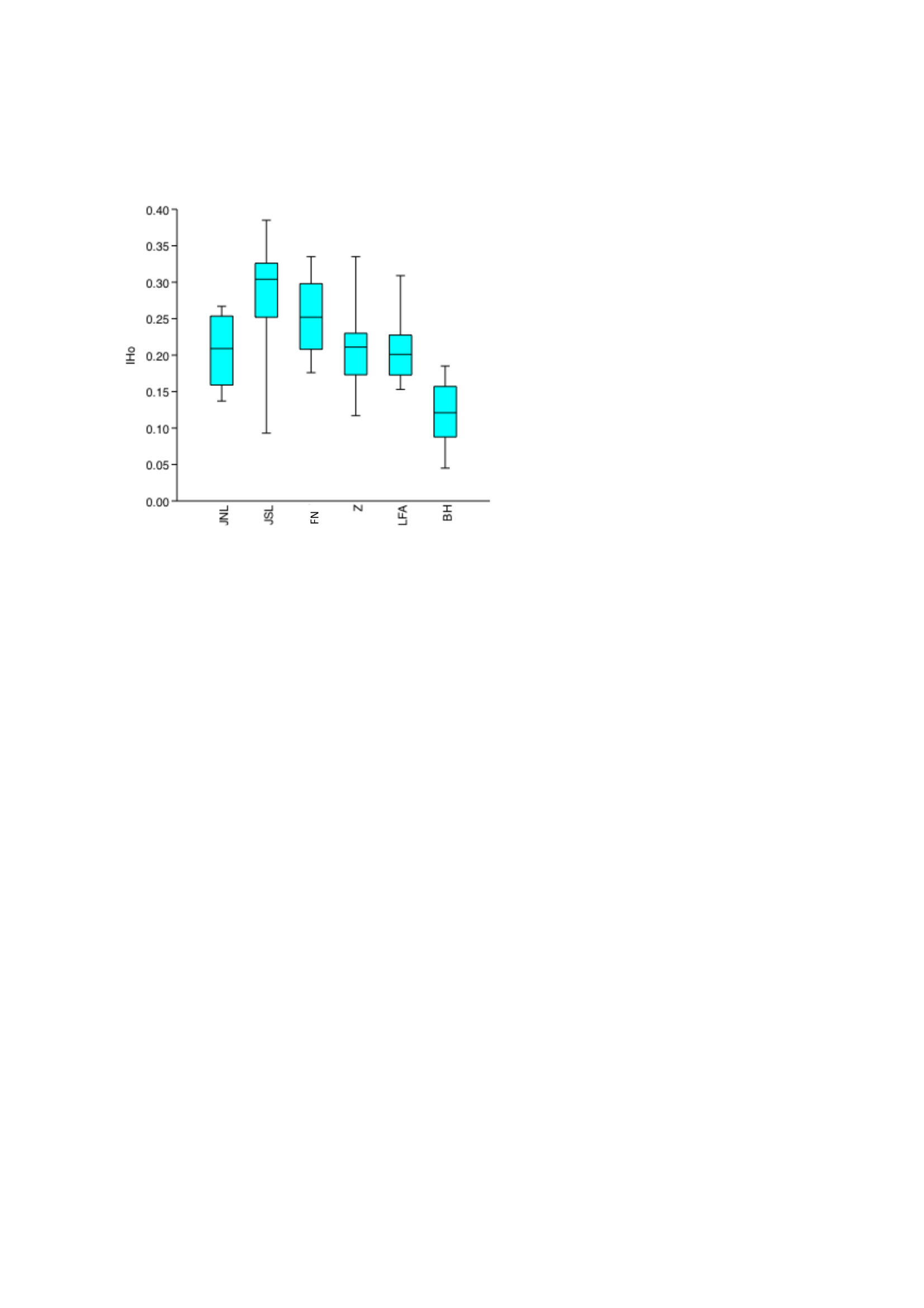

Supplement: S1 Fig — Box plot of the individual heterozygosity (iHO) estimated for the six populations: Jutland north of the Limfjord (JNL), Jutland south of the Limfjord (JSL), Funen (FN), Zealand (Z), Lolland and Falster (LFA) and Bornholm (BH). (TIFF) [file pone.0227205.s005.tiff]

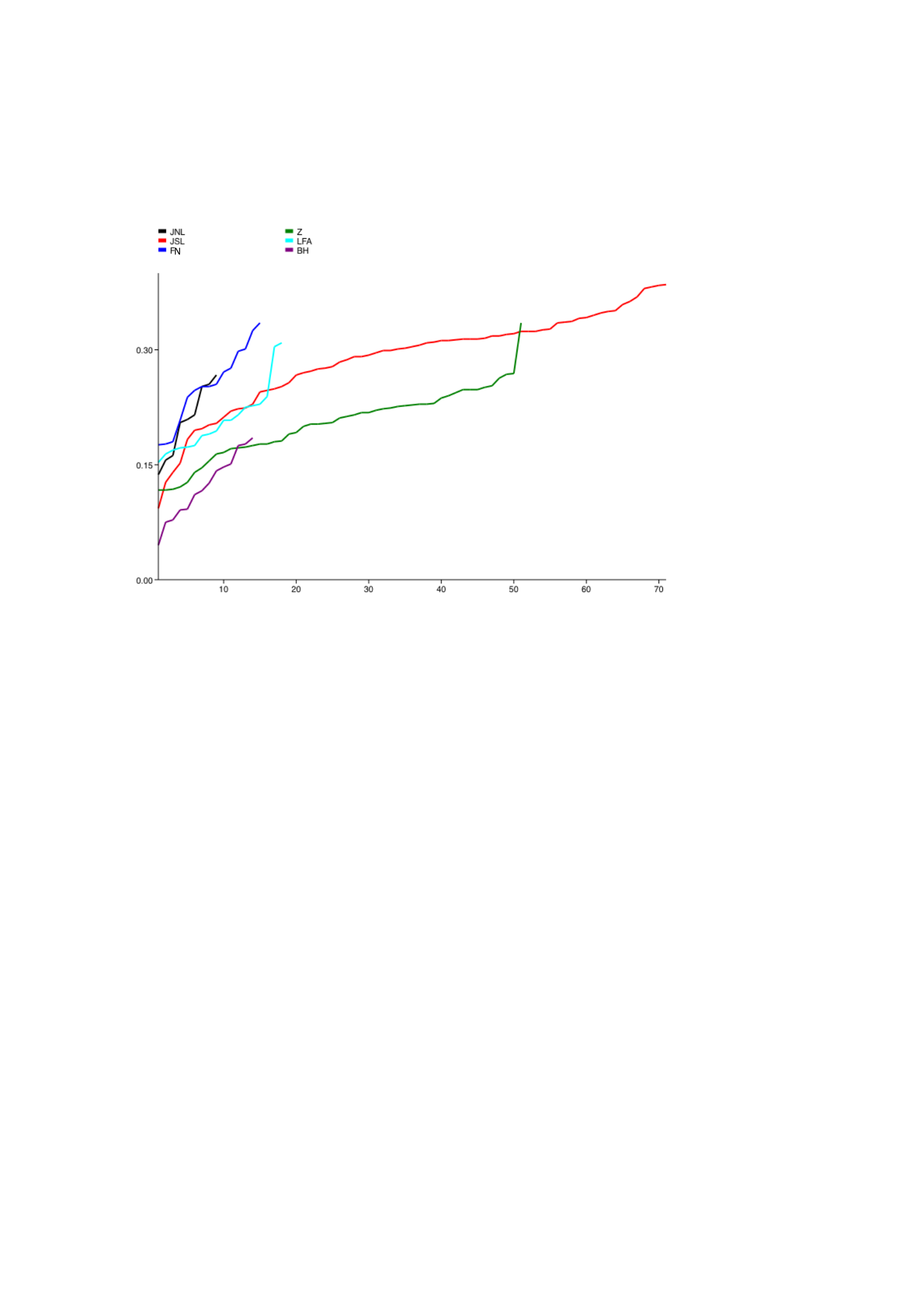

Supplement: S2 Fig — Plot of the iHO values for each of the six populations ranked from the lowest to the highest values within each population. (TIFF) [file pone.0227205.s006.tiff]

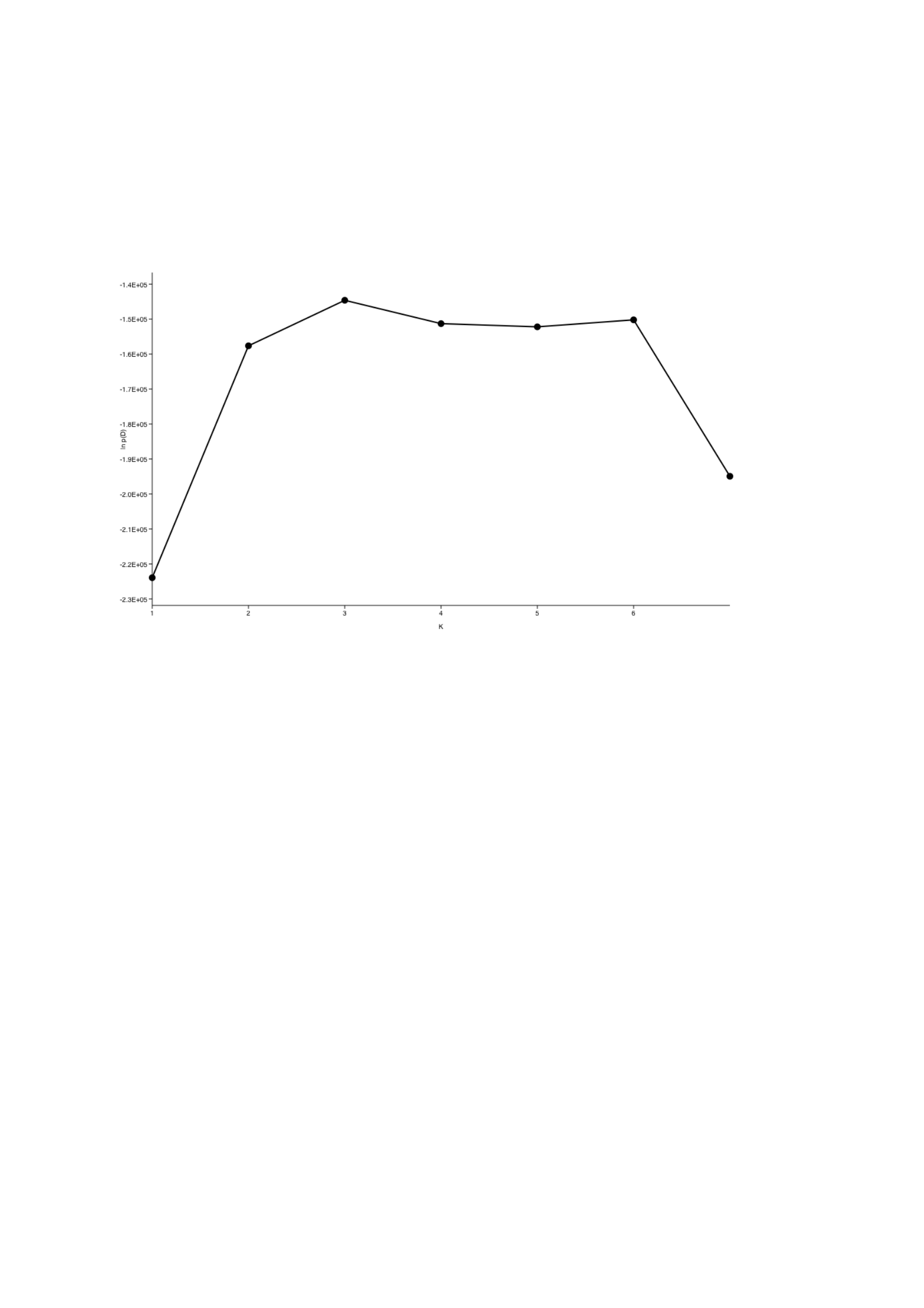

Supplement: S3 Fig — Likelihood plot of STRUCTURE results. Ln P(D) is the mean likelihood of K, the number of simulated clusters. The most likely K is that where ln P(D) is maximized. (TIFF) [file pone.0227205.s007.tiff]

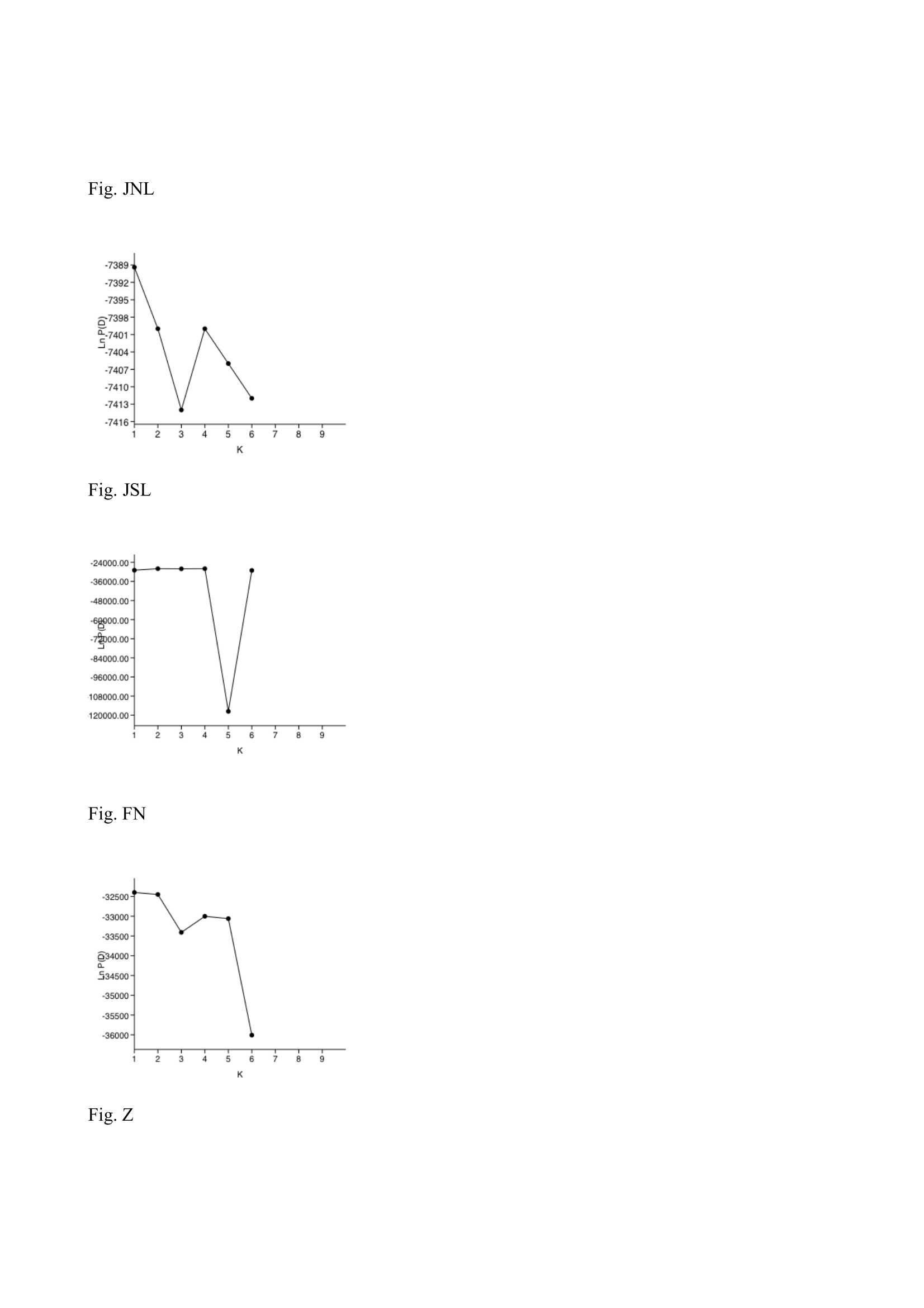

Supplement: S4 Fig — The figures are representing the Likelihood plots of results for each of the populations run separately in STRUCTURE (JNL, JSL, FN, Z, LFA, BH). Ln P(D) is the mean likelihood of K, the number of simulated clusters. The most likely K is that where ln P(D) is maximized. Jutland north of the Limfjord (JNL), Jutland south of the Limfjord (JSL), Funen (FN), Zealand (Z), Lolland and Falster (LFA) and Bornholm (BH). (TIFF) [file pone.0227205.s008.tiff]

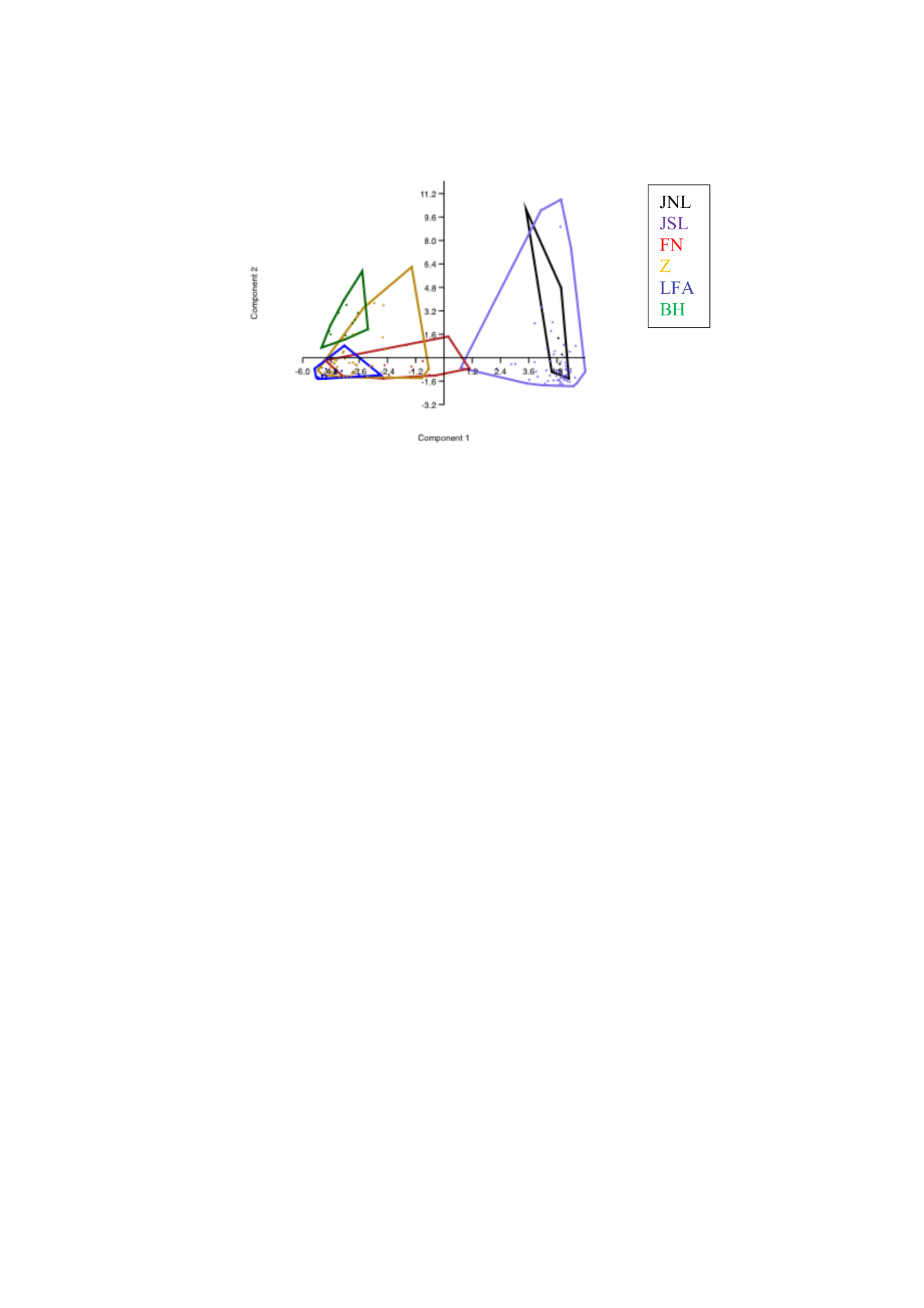

Supplement: S5 Fig — Principal Component Analysis of the PC1 (Eigenvalues 19.54; variance explained; 79.24%) and PC2 (Eigenvalues 5.12; variance explained; 20.76%) and the convex hulls. The following colours are indicating the six discovered populations: JNL: Jutland north of the Limfjord (Black); JSL: Jutland south of the Limfjord (Purple); FN: Funen (Red); Z: Zealand (Orange); LFA: Lolland and Falster (Blue); BH: Bornholm (Green). (TIFF) [file pone.0227205.s009.tiff]
